# Aloe vera gel for prevention of chemotherapy-induced hyperpigmentation: Four case reports

**DOI:** 10.1097/MD.0000000000034037

**Published:** 2023-06-23

**Authors:** Chia-Chi Chiu, Yi-Wen Hsiao, Yu-Chuan Wen, Tsung-Yen Chang, Shih-Hsiang Chen, Tang-Her Jaing

**Affiliations:** a Department of Nursing, Chang Gung Memorial Hospital, Taoyuan, Taiwan; b Division of Hematology and Oncology, Department of Pediatrics, Chang Gung Children’s Hospital, Chang Gung University, Taoyuan, Taiwan.

**Keywords:** Aloe vera gel, chemotherapy-induced hyperpigmentation, onconutraceutical

## Abstract

This study aimed to evaluate the efficacy of topical application of Aloe vera gel in preventing chemotherapy-induced hyperpigmentation (CIH). CIH is a common side effect of chemotherapy that causes skin irritation, redness, and itching. Aloe vera has been studied for its potential use in treating radiation-induced dermatitis, which may help alleviate some of the symptoms associated with this condition.

**Patient concerns:**

In this study, 4 children requiring curative chemotherapy were prospectively enrolled and treated with Aloe vera gel.

**Diagnosis:**

Acute skin reactions were monitored and classified according to the Common Terminology Criteria for Adverse Events Grading Scale.

**Interventions:**

Patients were asked to use the gel on one-half of the body field twice daily from the beginning of treatment until 4 weeks after the completion of chemotherapy, with no medication to be used on the other half.

**Outcomes:**

The results indicate that applying Aloe vera gel may reduce the visibility of hyperpigmentation at subsequent time points. The most important observation was that the continued application of Aloe vera gel 4 weeks after the completion of chemotherapy was effective in reducing the grading of CIH.

**Lessons:**

These effects highlight the potential of Aloe vera gel as a topical onconutraceutical treatment for CIH.

## 1. Introduction

Dermatologic adverse reactions to anticancer therapies can negatively influence dosing and quality of life (QOL). The type and severity of skin reactions can vary depending on the chemotherapy drug used, dosage, and sensitivity of the individual to these drugs.

Aloe vera is a juicy plant species belonging to the genus Aloe. It has anti-inflammatory and moisturizing properties, which can help soothe and hydrate skin. It can also help to reduce redness and irritation. Some studies have found that topical application of aloe vera gel to the affected area can help reduce the severity of radiation-induced dermatitis.^[1–3]^

Aloe vera is a herbal remedy with an established history of use. It has also been reported to exert protective effects against radiation-induced skin damage.^[4]^ Aloe vera gel has an extensive range of biological activities, including antifungal, anti-inflammatory, and hepatoprotective properties.^[5]^

Evaluation of Aloe vera in a randomized trial against the best clinical practice or placebo would have nonremovable ambiguities because the best clinical practice is unknown or mostly agreed on and because even a placebo may also affect radiation skin reactions through its moisturizing or other properties.^[1]^ Considering the aforementioned factors and uncertainties regarding using Aloe vera to prevent chemotherapy-induced hyperpigmentation (CIH), we decided to examine this issue in a self-controlled clinical trial.

## 2. Materials and methods

### 2.1. Data source

Accurate diagnosis and appropriate management of chemotherapy-related adverse effects require clinicians to know the most common skin reaction patterns for the drugs that patients receive. All study procedures were conducted at the Chang Gung Memorial Hospital. Informed consent was obtained before the inclusion of patients. Requests for approval to access groups under 18 years of age were assessed on a case-by-case basis by the Institutional Review Board. Patients must be conscious, awake, and able to understand and answer fluent questions in Chinese.

The hyperpigmentation grade was evaluated using the Common Terminology Criteria for Adverse Events v.5.0. Skin hyperpigmentation scored as grade 1 = “hyperpigmentation covering < 10% body service area; no psychological impact”; grade 2 = “hyperpigmentation covering > 10% body service area; psychological impact.”

For the intervention, a commercially available Aloe vera gel was provided to patients with CIH, who were asked to use the gel on only one-half of the body field twice daily from the beginning of treatment until 4 weeks after completion of chemotherapy, with no medication to be used on the other half. The gel provided to the patients included Aloe vera in addition to lanolin oil, glyceryl stearate, diluted collagen, tocopherol, allantoin, and paraben. In the case of symptomatic CIH, the treatment routine of topical corticosteroids was prohibited over the entire treatment area. The dorsal and palmar surfaces of 1 hand were applied, whereas the other hand served as the control.

The aim of using Aloe vera gel to prevent CIH is to reduce the severity and duration of skin reactions caused by cancer treatment, improve patients’ QOL, and minimize the need for additional medications to manage skin-related side effects.

### 2.2. Trial oversight

The trial was approved by the Ethics Committee of Chang Gung Memorial Hospital and followed the Good Clinical Practice guidelines of the International Council for Harmonization and the provisions of the Declaration of Helsinki. All parents or guardians of patients provided written informed consent. In compliance with the regulations of the Institutional Review Board (202100082A3), we prospectively analyzed the data. Informed consent was obtained from the patient for publication of this case report details (see online Supplementary data S1, Supplemental Digital Content, http://links.lww.com/MD/J138).

## 3. Results

**Figure 1.**
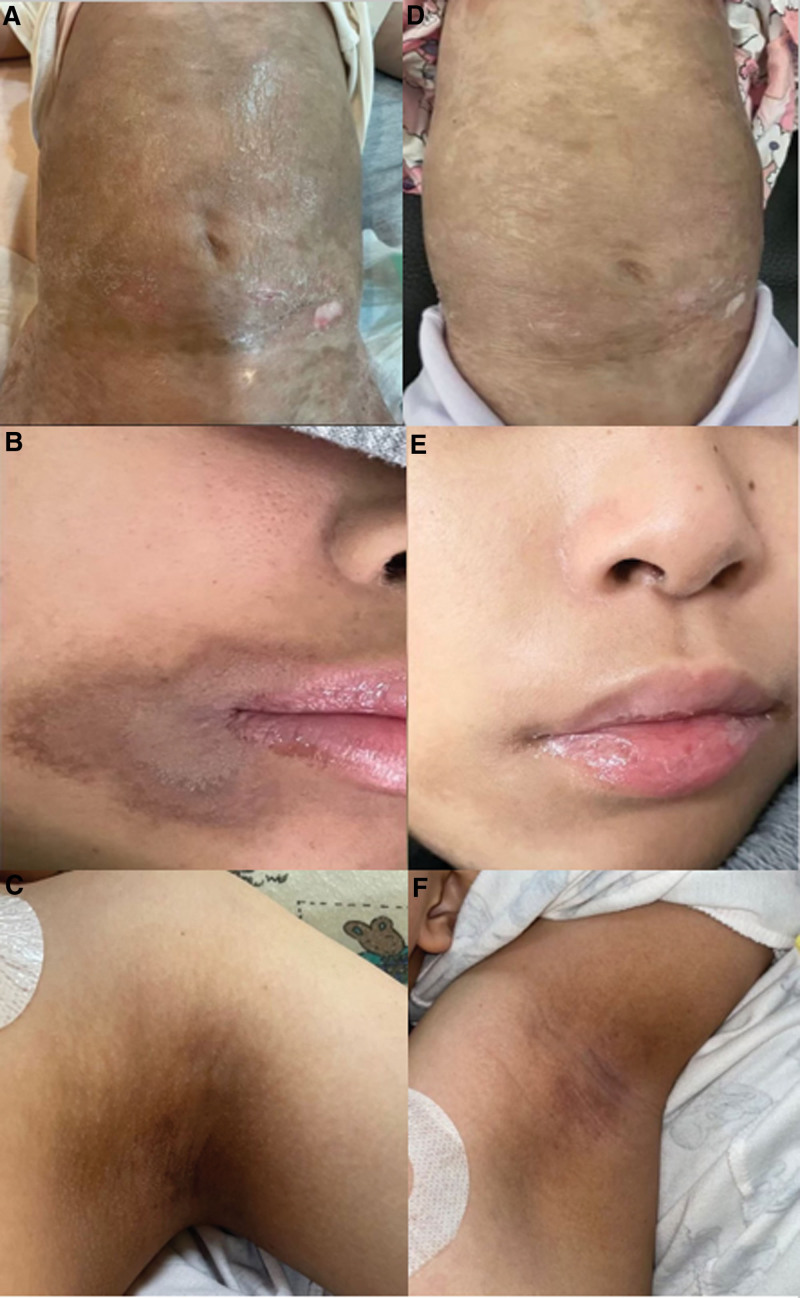
Right: Baseline hyperpigmented macules in 3 patients. Left: After 4 weeks of treatment with Aloe vera gel.

**Figure 2.**
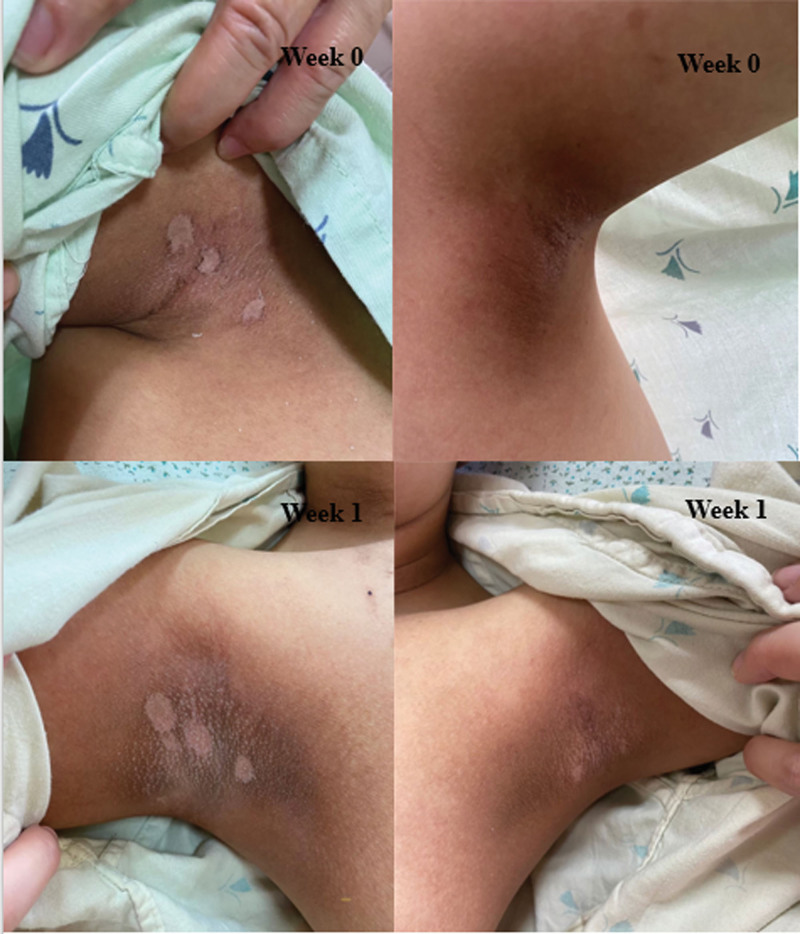
The photographs of one patient show the efficacy of Aloe vera gel: the experimental side (right) compared with the control side (left) between week 0 and week 1.

Although research has not been extensive, there are encouraging signs that they can effectively fade post-chemotherapy hyperpigmentation (Fig. 1). The half applied with Aloe vera gel showed reduced grade severity of hyperpigmentation compared to the other half that had not been applied (Fig. 2). It should be noted that hyperpigmentation can be induced by some chemotherapy agents. Therefore, the tumor may not disappear if the patient continues to receive chemotherapy.

Although Aloe vera is commonly used to treat various skin conditions, there is limited scientific evidence to support its use, specifically for the prevention of CIH. Aloe vera is supposed to work by moisturizing and cooling the skin, reducing inflammation, and promoting the growth of new skin cells. At the end of the study, the patients were satisfied with their skin caring outcomes.

## 4. Discussion

However, classic chemotherapy is often associated with adverse effects. The most common side effect is dermatological.^[6]^ Some chemotherapy agents, including ifosfamide, cyclophosphamide, daunorubicin, bleomycin, busulfan, 5-fluorouracil, platinum-based agents, and thiotepa, can cause and exacerbate hyperpigmentation, which can impair QOL.^[7,8]^ It can be localized, diffuse, or have a distinctive pattern.^[9,10]^

Phytochemicals are natural compounds extracted or derived from plants and have been reported for skin hyperpigmentation treatment owing to various mechanisms that inhibit melanogenesis. Aloesin, a glycoprotein extracted from Aloe vera, was reported to exhibit antityrosinase activity in a dose-dependent manner. It works by inhibiting L-DOPA oxidation and has shown better affinity than kojic acid, arbutin, etc.^[11]^ Aloe Vera has been used to treat eczema, skin burn, frostbite, and other dermatologic diseases for many years.^[12]^ While there is little scientific evidence that Aloe vera can reduce the size of hyperpigmented spots, some studies have reported that it works to lighten dark spots.^[11]^

Post-chemotherapy hyperpigmentation can be a temporary or long-term side effect of chemotherapy, depending on the individual and the drugs used for treatment.^[13]^ In most cases, hyperpigmentation fades over time; however, it can take several months or even years for the skin to return to its normal color. However, this study examined an alternative option to solve the problem of hyperpigmentation affecting the QOL.

## 5. Conclusion

While Aloe vera gel may benefit the skin, more research is needed to determine its effectiveness in treating CIH. Although there are various indications for its use, randomized controlled trials are the best method for determining its efficacy.

## Acknowledgments

We thank the patients for participating in the study and the colleagues for their recruitment and interviews.

## Author contributions

**Conceptualization:** Chia-Chi Chiu, Tang-Her Jaing.

**Data curation:** Yi-Wen Hsiao.

**Formal analysis:** Chia-Chi Chiu, Yu-Chuan Wen.

**Investigation:** Shih-Hsiang Chen, Tang-Her Jaing.

**Methodology:** Chia-Chi Chiu, Tsung-Yen Chang.

**Supervision:** Yu-Chuan Wen.

**Validation:** Shih-Hsiang Chen, Tang-Her Jaing.

**Writing – original draft:** Chia-Chi Chiu, Yi-Wen Hsiao.

**Writing – review & editing:** Tang-Her Jaing.

## Supplementary Material


